# Cardiotoxicity of Intravenously Administered CdSe/ZnS Quantum Dots in BALB/c Mice

**DOI:** 10.3389/fphar.2019.01179

**Published:** 2019-10-08

**Authors:** Li Li, Jinglin Tian, Xiaomei Wang, Gaixia Xu, Wenxiao Jiang, Zhiwen Yang, Dongmeng Liu, Guimiao Lin

**Affiliations:** ^1^Department of Physiology, School of Basic Medical Sciences, Health Sciences Center, Shenzhen University, Shenzhen, China; ^2^Key Laboratory of Optoelectronics Devices and Systems of Ministry of Education/Guangdong Province, College of Optoelectronic Engineering, Shenzhen University, Shenzhen, China; ^3^National-Regional Key Technology Engineering Laboratory for Medical Ultrasound, Guangdong Key Laboratory for Biomedical Measurements and Ultrasound Imaging, Department of Biomedical Engineering, School of Medicine, Shenzhen University, Shenzhen, China

**Keywords:** CdSe/ZnS quantum dots, cardiotoxicity, *in vivo*, nanotoxicity, echocardiography

## Abstract

Since CdSe quantum dots (QDs) are increasingly used in electronics, medical, and pharmaceutical science due to their excellent optical properties, it is necessary to carry out thorough and systematic studies on their biosafety. Numerous studies have reported the toxicity of QDs on liver, kidney, immune system, and reproductive system. However, few studies have been done on the cardiotoxicity of QDs. In this study, we administered carboxylated CdSe/ZnS QDs in BALB/c mice *via* the tail vein and analyzed the *in vivo* cardiotoxicity of CdSe/ZnS QDs. The body weight, hematology, serum biochemistry, histology, heart elements concentration, echocardiography, and heart oxidative stress markers were carried out at different time. There were no significant differences in body weight and heart organ index between QDs group and the control group. Hematology results showed the platelet (PLT) counts on Day 1 and Day 42 in both high dose QDs group and low dose QDs group, and the PLT counts on Day1 in the high dose group were significantly higher than that in control group. Serum biochemistry results showed that lactate dehydrogenase (LDH), creatine kinase (CK), and creatine kinase isoenzyme (CK-MB) of mice exposed to CdSe/ZnS QDs were significantly higher than that of the control group on Day 1, and CK-MB levels still remained high on Day 7. A higher concentration of Cd was observed in the heart of CdSe/ZnS QDs exposed mice on Day 42, whereas no Cd was detected in the control group, which suggested that QDs can accumulate in heart. No significant histopathological changes and cardiac function were observed in all mice at different time after treatment. Increased level of glutathione peroxidase (GPx) and malondialdehyde (MDA) was observed in mice administered with high dose QDs on Day 1, and increased level of total antioxidant capacity (T-AOC) and MDA activities was observed on Day 42. These results indicated that CdSe/ZnS QDs could accumulate in heart, cause some biochemical indicators change, induce oxidative damage, and have cardiotoxicity. Our findings might provide valuable information on the biological safety evaluation of the cardiovascular system of QDs.

## Introduction

Quantum dots (QDs), also known as semiconductor nanocrystals, are a class of novel nanomaterials with diameter ranges from 1 to 10 nm. These nanocrystals are generally spherical or quasi-spherical and contain of elements belonging to group II-VI (CdSe, CdS, CdTe, ZnS, et al.) or group III-V (InP, InAs, Cu_2_InS, et al.)(Mattoussi et al., 2000; Brunetti et al., 2013). QDs have photoluminescence properties. The fluorescence emission wavelength of QDs covers the whole visible region, even the infrared region, if adjusting their size and composition. Compared with traditional fluorescent dyes, QDs have larger Stokes shift, good photobleaching resistance, high quantum yield, strong, and stable fluorescence intensity and are appropriate for long-term or real-time observation under continuous excitation of exciting light. As a semiconductor nanomaterial, QDs have many unique optical properties, such as quantum size effect, surface effect, dielectric confinement effect, and quantum tunneling effect (Lin et al., 2018). So far, QDs have been extensively used in the fields of light emitting devices, solar battery, catalysis, fluorescence imaging, drug delivery, and therapeutic functionalities (Lee and Lo, 2009; Wang and Chen, 2011; Li et al., 2017). However, concerns have been raised that the widely used of QDs may accumulate in the environment, affect surrounding microbiota, and challenge to human health (Reshma and Mohanan, 2019).

As the potential toxicity of QDs has brought great controversy to its wide application, numerous toxicological studies on QDs have been carried out for this purpose. It is common knowledge that the toxicity of QDs is related to many factors, such as chemical composition, particle size, shape-structure, charge, surface modification, and concentration (Manshian et al., 2017; Zheng et al., 2017; Murase et al., 2019). For example, Hu et al. reported that small negative charged CdSe/ZnS QDs showed greater cytotoxic than larger negatively charged CdSe/ZnS QDs in P. chrysosporium (Hu et al., 2017). Meanwhile, some published reports have provided the toxicity and mechanism of QDs in different cellular models. In vitro, QDs can induce cytotoxicity in multiple cell lines, contains HepG2, HCC-15, Hela, and PC-12, mainly in the form of reduced cell viability, generating reactive oxygen species (ROS), DNA damage, mitochondrial damage, or cell death (Chen et al., 2018; Duong et al., 2018). The mechanism of toxicity induced by QDs may relate to the release of the metal ion, oxidative stress, and inflammation (Liu et al., 2017; Lu et al., 2019). Although in vitro experiments can preliminarily assess the toxicity and mechanism of QDs, these in vitro models cannot fully match the complex biological interactions occurring in vivo. In this regard, some in vivo studies have reported several effects of exposure of QDs (Xu et al., 2016). Rotomskis et al. reported that carbonylated CdSe/ZnS QDs could cause the fish embryos damage and induce the development of fish (Rotomskis et al., 2018). Zhang et al. reported that CdSe QDs could damage the placenta and lead to fetus malformation though the QDs were effectively blocked by the placental barrier (Zhang et al., 2016). Moreover, some in vivo studies have demonstrated that QDs can induce morphological and functional impairments in the liver, lung inflammation, and injury (Roberts et al., 2013; Yang et al., 2014; Lu et al., 2016). Nevertheless, understanding of QDs toxicity is still poor and the underlying mechanism is particularly unclear.

Heart is the principal organ to promote blood circulation in the whole body. When QDs enter the bloodstream, they first come into contact with blood, and then will travel back to the heart by the vein. In order to systematic evaluate the biocompatibility of QDs, it is also important to study the cardiotoxicity of QDs. However, at present, in vivo toxicity studies of QDs mainly focus on the effects in liver, kidney, spleen, and reproductive system. There were no reports on the evaluation of cardiotoxicity of QDs as far as we know. Hence, in this study, we systematically investigated the cardiotoxicity of the most widely used CdSe/ZnS QDs. We monitored the survival status of mice after intravenous injection of QDs, detected the hematology, serum biochemistry, histology, heart elements concentration, and echocardiography, and analyzed the cardiotoxicity mechanism induced by QDs from the perspective of oxidative stress. Our findings provided new experimental data for better and more comprehensive understanding of the biological safety of QDs.

## Materials and Methods

### Instrumentation, Materials, and Reagents

F-4600 fluorescence spectrophotometer (HITACHI, Japan); eta potential and particle size analyzer (Brookhaven Instruments Inc., USA); general purpose UV/Vis spectrophotometer (Beckman Coulter Inc., USA); transmission electron microscope (TEM) (JEM-1230, NIPPON TEKNO, Japan); paraffin embedding station (Leica, German); tissue dehydrator (Leica, German); ultra-thin semiautomatic microtome (Leica, German); mass spectrometer (Agilent, USA); fully automatic five-classification hematology analyzer (Shenzhen Dimai Biotechnology Co., Ltd., China); automatic blood biochemical analyzer (Mindray medical international limited, China); Axio observer fluorescence microscope (ZEISS, Germany); full automatic microwave digestion instrument (LabTech, China); and inductively coupled plasma mass spectrometry (ICP-MS, PerkinElmer, USA).

CdSe/ZnS QDs (Thermo Fisher Scientific, USA); HE staining solution (Biosharp, China); tissue fixative (Wexis, China); blood biochemical detection reagents (Mindray medical international limited, China); blood routine reagents (Shenzhen Dimai Biotechnology Co., Ltd., China); and protein quantitative kit (Thermo Fisher Scientific, USA). Catalase (CAT) assay kit, glutathione reductase (GR) detection kit, lipid peroxidation (malondialdehyde, (MDA)) detection kit, total antioxidant capacity (T-AOC) test kit, glutathione peroxidase (GPx) detection kit, and total superoxide dismutase (SOD) assay kit were purchased form Beyotime Biotechnology co., ltd, China.

### Characterization of CdSe/ZnS QDs

CdSe/ZnS QDs (Product No. Q21321MP) were purchased from Thermo Fisher Scientific, USA. The CdSe/ZnS QDs was coated with a polymer layer and terminated with -COOH surface groups. Morphology images of CdSe/ZnS QDs were obtained by JEM-1230 TEM with an acceleration voltage of 200 kV. The hydrodynamic size distribution and zeta potential of CdSe/ZnS QDs were obtained by zeta potential and particle size analyzer. Fluorescence spectrum and absorption spectrum of CdSe/ZnS QDs were obtained by F-4600 fluorescence spectrophotometer and UV/Vis spectrophotometer, respectively.

### Animal Husbandry and Treatment

Healthy male BALB/c mice aged 6 weeks old were purchased from the Medical Laboratory Animal Center of Guangdong Province. All mice were housed in independent ventilation cages in the animal room (relative humidity at 60 ± 10% and a 12-h light/dark circle). Room temperature was maintained at 24 ± 2°C. Distilled water and sterilized food were available *ad libitum*. The animal study protocols were conducted in accordance with the Guide for the Care and Use of Laboratory Animals Center of Shenzhen University and approved by the Experimental Animal Ethics Committee of Shenzhen University (Permit No. 201412012).

Animals were randomly divided into three groups: high dose group (2 nmol/kg BW of CdSe/ZnS QDs), low dose group (0.2 nmol/kg BW of CdSe/ZnS QDs), and control group (treated with PBS buffer solution). After the CdSe/ZnS QDs was diluted with PBS buffer solution, a single injection of CdSe/ZnS QDs was administered through the tail vein with the volume of 200 μl per mouse. Mice in the control group were given the equal volume of PBS buffer solution only. Body weight, behavior, and mortality were monitored and recorded carefully after treatment.

### Necropsy and Sample Collection

At predetermined time points (1, 7, 14, 28, and 42 days) after treatment, seven mice from each group were anesthetized with isoflurane. Blood samples of mice were collected from the posterior orbital venous plexus. 50 μl of whole blood for routine blood tests was collected in anticoagulant tube. The rest of the whole blood for blood biochemical analysis was collected in procoagulant tube. Serum was obtained by centrifugation at 4,000 rpm for 10 min and then stored at -20°C until used.

Then, the animals were sacrificed by cervical dislocation. The hearts were excised and weighed accurately. Then, the organ weight/BW coefficient of the heart was calculated as organ weight (wet weight, mg)/BW (g) × 100%. A small piece of tissue was dissected, fixed in tissue fixative, and stored at 4°C until used.

### Hematology Analysis

The hematology parameters in blood were detected by fully automatic five-classification hematology analyzer. The specific hematology indexes in this study included white blood cell (WBC) count, red blood cell (RBC) count, and platelet (PLT) count.

### Serum Biochemical Analysis

The serum biochemical parameters were detected by automatic blood biochemical analyzer. The specific biochemical indexes in this study included lactate dehydrogenase (LDH), creatine kinase (CK), and creatine kinase isoenzyme (CK-MB).

### Determination of Elements Concentration

The hearts from each mouse were prepared and digested in the microwave digestion instrument by adding 4 ml 65% nitric acid (HNO_3_) and 1 ml 30% hydrogen peroxide (H_2_O_2_). ICP-MS was utilized to quantify the amount of cadmium (Cd) and selenium (Se) in the tissue. For all measurements, nitric acid blank, Cd standards, and Se standards were prepared and tested concurrently with test samples. Distribution of Cd or Se in tissues was calculated using the following equations: [Cd or Se] concentration (μg/g wet tissue) = [Cd or Se] tissue suspension/wet weight of tissue.

### Histopathological Examination

The fixed tissues were dehydrated with different gradient concentrations of alcohol and then embedded in paraffin blocks. 5 μm thickness tissue sections were prepared and placed onto glass slides. After staining with hematoxylin and eosin (H&E), histopathological morphology was evaluated under the microscope by an independent pathologist unaware of the treatment.

### Cardiac Function Measurement

On Day 1 and Day 42 after treatment, mice in high dose group were lightly anesthetized with 1% isoflurane, removed chest hair with the depilatory, and echocardiographic examination of all mice was detected by Vevo2100 ultrasound imaging system. The measured parameters and the automatic calculation parameters of the ultrasound instrument included left ventricular anterior wall thickness (LVAW), left ventricular internal diameter (LVID), left ventricular posterior wall thickness (LVPW), left ventricular volume (LVV) both in end-systolic and end-diastolic, ejection fraction (EF), shortening fraction (FS), and left ventricular mass (LV MASS).

### Oxidative Stress Markers Detection

The levels of CAT, T-AOC, MDA, GPx, GR, and SOD in the heart tissues were estimated by the corresponding kit. CAT activity was measured by a colorimetric assay and results were expressed as U/mg protein. T-AOC was measured by 2, 2’-azino-bis(3-ethylbenzothiazoline-6-sulfonic acid) (ABTS) method. Trolox was used as the standard, and T-AOC of samples was represented by Trolox-equivalent antioxidant capacity, and results were expressed as μmol/mg protein. The MDA levels were determined by thiobarbital method, absorbance of red reaction products was measured at 532 nm, and results were expressed as μmol/mg protein. GPx activity was determined by quantifying the rate of oxidation GSH to GSSG. GR activity was measured at 412 nm using chromogenic substrates 5,5′-dithio-bis-[2-nitrobenzoic acid] (DTNB). SOD activity was determined by the method of WST-8. All results were expressed as U/mg protein.

### Statistical Analysis

Statistical analysis were carried out using SPSS 22.0 statistic software package. All data were expressed as mean ± standard deviation (SD). The difference between two groups was analyzed by two independent samples *t*-test. The difference among the three groups was assessed using one-way ANOVA. The differences were considered statistically significant if *P* < 0.05.

## Results

### Characterization of CdSe/ZnS QDs

**Figure 1:**
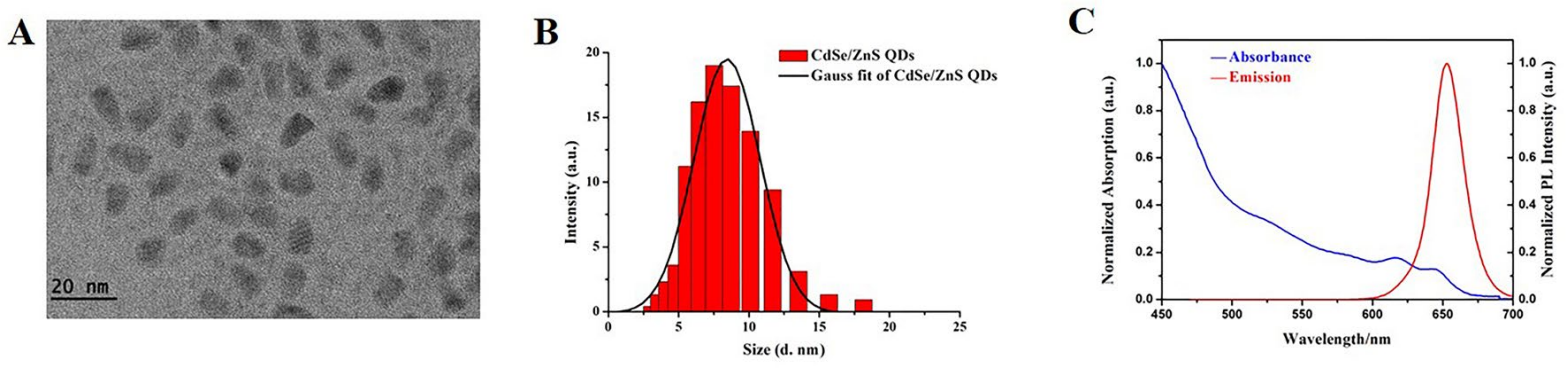
Characterization of CdSe/ZnS QDs. **(A)** TEM image of CdSe/ZnS QDs (Scale bar: 20 nm). **(B)** DLS of CdSe/ZnS QDs dispersed in deionized water. **(C)** Normalized absorption spectra (blue line) and normalized PL emission spectra (recorded at red line, λ*ex* = 450 nm) of CdSe/ZnS QDs, recorded at room temperature in a 1 cm quartz cuvette.

The morphology, particle diameter, and optical properties of CdSe/ZnS QDs were characterized and illustrated in **Figure 1**. Image of CdSe/ZnS QDs was captured by TME (**Figure 1A**) and it demonstrated that the CdSe/ZnS QDs were ellipsoid, with the diameter (6.60 ± 1.16) nm (minor axis) × (10.08 ± 1.15) nm (major axis). The CdSe/ZnS QDs were well dispersed in solution. The average hydrodynamic diameter was about (8.78 ± 1.29) nm (**Figure 1B**) and the zeta potentials were -(32.2 ± 2.07) mV, which were characterized by Zeta potential and particle size analyzer. Moreover, we also characterize the absorption spectra and photoluminescence (PL) emission spectra of the QDs. The absorption spectra were shown in **Figure 1C** (blue line) and the first exciton peak of the QDs was about at 650 nm. The PL spectra of the CdSe/ZnS QDs were shown in **Figure 1C** (red line) and there were narrow and symmetrical emission spectra at around 655 nm following excitation at 450 nm. The above results showed that the CdSe/ZnS QDs purchased in our study have uniform size, good stability, and good optical properties.

### Body Weight and Organ Weight/BW Coefficients

The dietary patterns and physical activity of the two group mice were observed after treatment. The mice treated with CdSe/ZnS QDs showed decreased food, water intake, and physical activity than the control group the next day, but then recovered in a few days later. No mice died both in the experimental group and the control group up to the 42^nd^ day.

**Figure 2:**
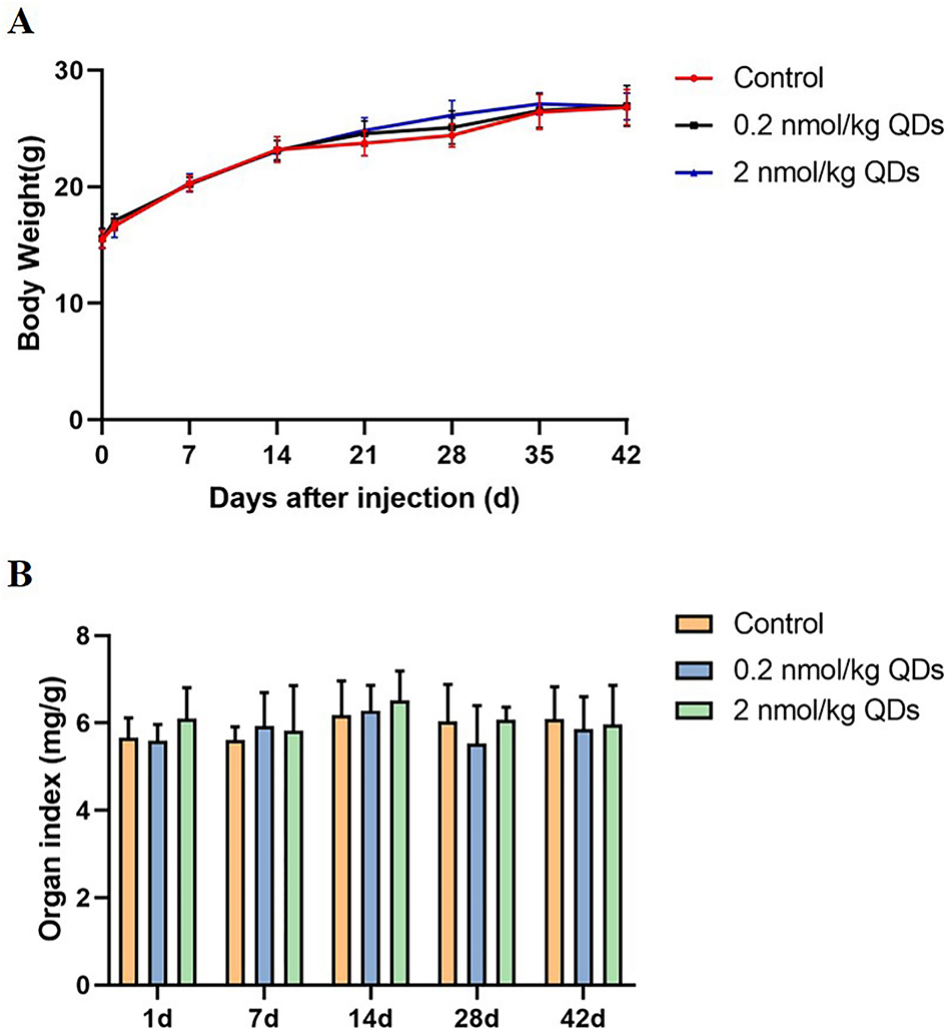
Body weight and organ weight/BW coefficients in mice. **(A)** The body weight curve of mice measured continuously for 42 days. **(B)** The heart weight/BW coefficients of mice.

Body weight and organ weight/BW coefficient are two basic indexes for evaluating toxicity of nano-materials to mice. Continuous measurement of mice’s body weight could reflect its growth, development, and health status. The body weight of mice was continuously monitored for 42 days and the data was shown in **Figure 2A**. No significant difference was observed between CdSe/ZnS QDs treated group and the control group. After sacrificing the mice on Day 1, Day 7, Day 14, Day 28, and Day 42, the hearts were weighed and the heart organ index was calculated. Our results showed there was no significant difference between QDs group and the control group (**Figure 2B**).

### Hematology Analysis

**Figure 3:**
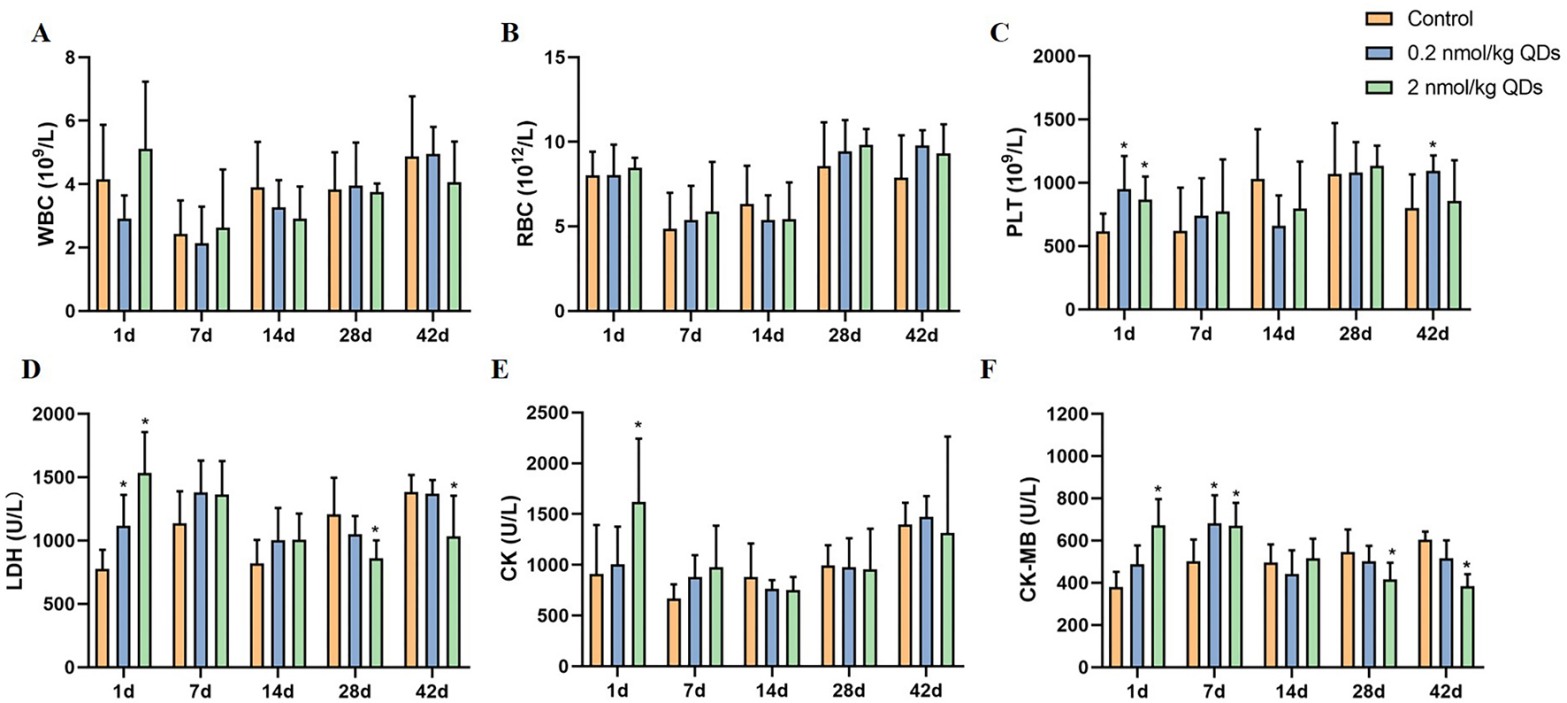
Effects of CdSe/ZnS QDs on hematological parameters in blood **(A–C)** and biochemical parameters in serum **(D–F)**. **(A)** WBC count. **(B)** RBC count. **(C)** PLT count. **(D)** LDH level. **(E)** CK level. **(F)** CK-MB level. (*Significantly different compared to control *P* < 0.05.)

When mice were exposed to CdSe/ZnS QDs through intravenous injection, QDs first contacted with the cells and components of blood. It is necessary to detect the changes of blood cells. In this study, blood routine of mice at different time after exposure was carried out and the results were shown in **Figure 3 (A–C)**. The levels of PLT on Day 1 and Day 42 in both high dose QDs group and low dose QDs group and the levels of PLT on Day1 in the high dose group were significantly higher than that in control group (*P* < 0.05). No significant differences were found in levels of WBC and RBC in CdSe/ZnS QDs treated mice as compared to the control group.

### Serum Biochemical Analysis

Cardiovascular system was required for the systemic distribution of QDs when QDs was injected into mice through the tail vein. Detection serum biochemical indicators related to cardiac function can reflect the adverse effects of QDs on heart. LDH, CK, and CK-MB in serum were measured and the results were showed in **Figure 3 (D–F)**. On the first day after being exposed to QDs, the levels of LDH, CK, and CK-MB in QDs exposed group were significantly higher than that in the control group. CK-MB levels in QDs exposed group were still higher than that in control group on Day 7, whereas there were no significant differences of CK and LDH among the three groups. It suggested that QDs are somewhat toxic to the heart after exposure.

### Elements Concentration in Heart

**Figure 4:**
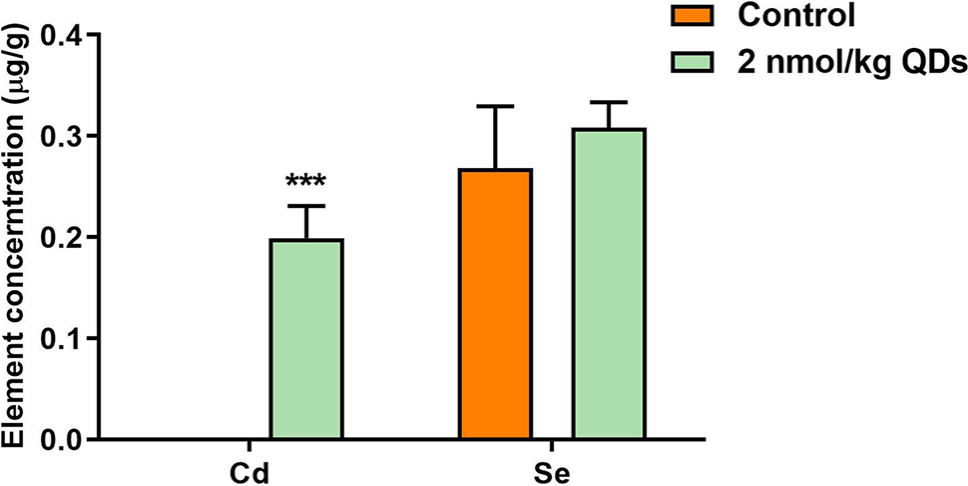
The concentration of Se and Cd in the heart of mice on the 42^nd^ after treatment. (***Significantly different compared to control *P* < 0.001.)

After injected into the caudal vein of mice, QDs could be transported to various organs by the blood circulatory system and then metabolized and excreted. Meanwhile, QDs may accumulate in multiple organs. Measuring the level of unique and principal elements of QDs can reflect the accumulation and excretion of QDs. In our study, the levels of Se and Cd in heart were determined by ICP-MS and the results were presented in **Figure 4**. On the 42^nd^ day after treatment, Cd could be detected in the heart of mice treated with CdSe/ZnS QDs, which was significantly higher than that in the control group. It indicated that CdSe/ZnS QDs could accumulate in heart and could not be completely excreted up to 42 days. However, QDs have a low accumulation in the heart. As Se is one of the essential trace elements in the body and less QDs accumulated in the heart, there was no significant difference of Se concentration in the heart between the CdSe/ZnS QDs group and the control group. The results above illustrated that CdSe/ZnS QDs can accumulate in the heart of mice up to 42 days, but the accumulative concentration was very low.

### Histopathological Detection

**Figure 5:**
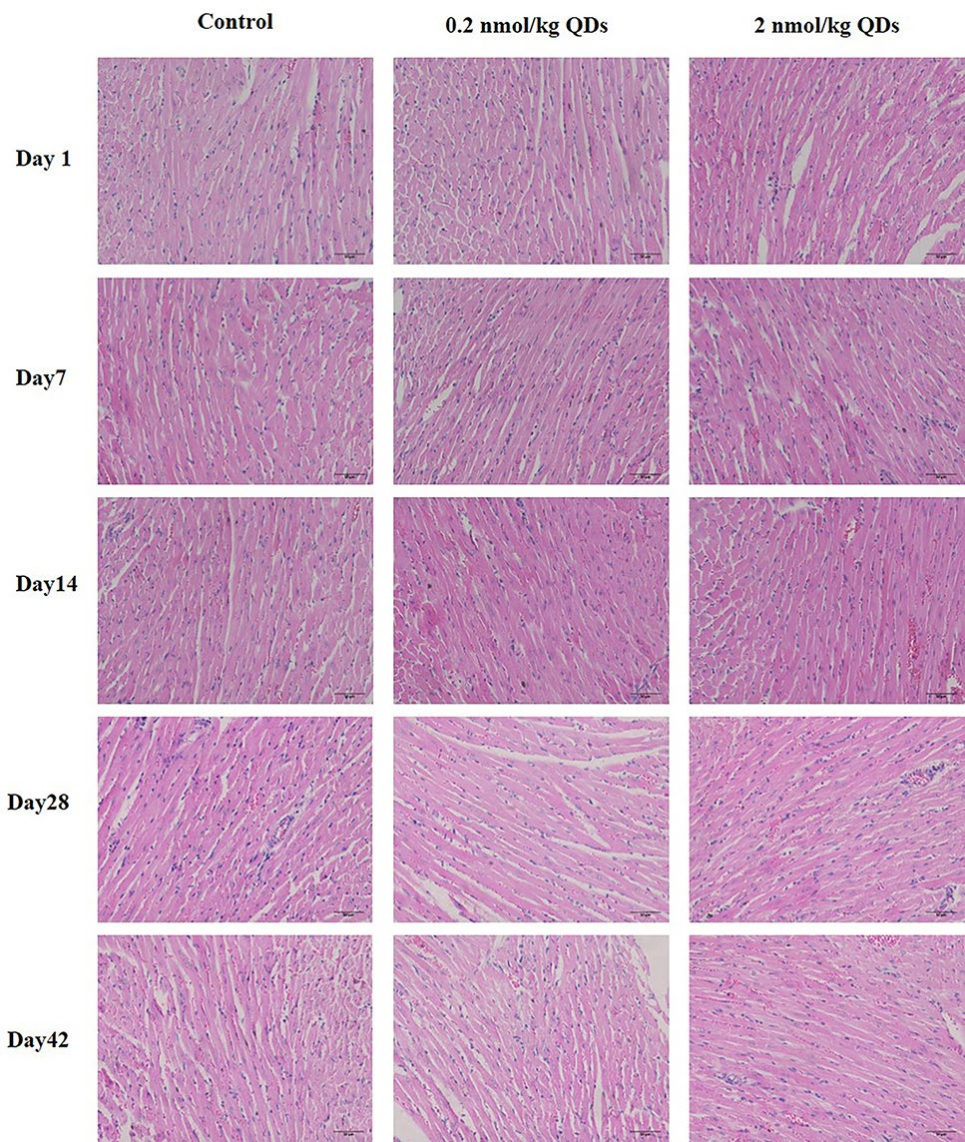
H&E staining of heart from QDs-treated or control mice (scale bar: 50 µm).

As CdSe/ZnS QDs could accumulate in heart, H&E staining was used to detect the histopathological changes in the heart tissue. The results were shown in **Figure 5**. No obvious histopathological changes of heart were observed from mice at different time points. It suggested that CdSe/ZnS QDs may accumulate in the heart for up to 42 days and there were no significant histopathological changes in the heart of mice.

### Cardiac Function Measurement

**Figure 6:**
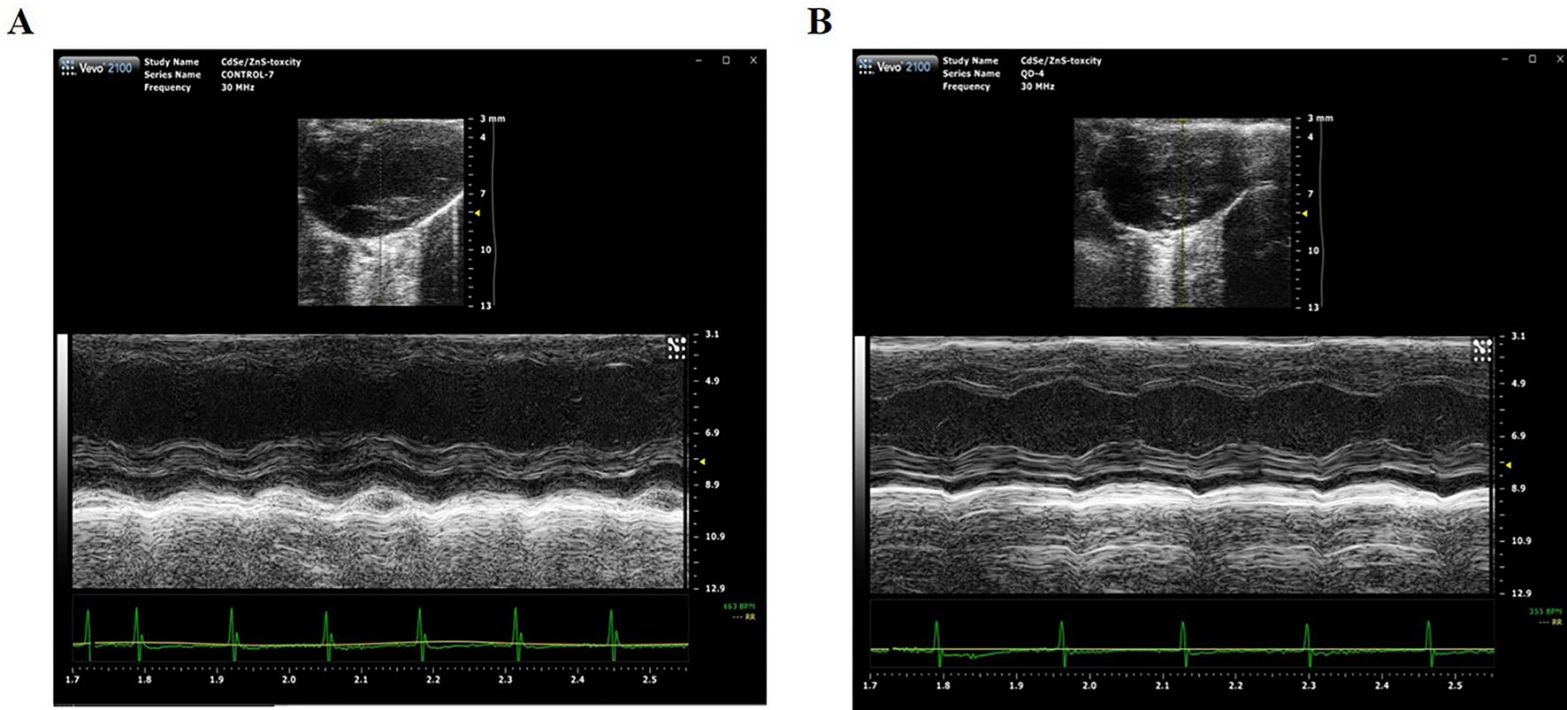
Cardiac function measurement detected by Vevo2100 ultrasound imaging system. **(A)** Echocardiography of mice in control group. **(B)** Echocardiography of mice treated with CdSe/ZnS QDs.

Echocardiography is the use of ultrasonic to detect the periodic activity of the heart wall, ventricle, and valves, which can be used to reflect the changes in cardiac function. The echocardiogram of mice was measured by small animal ultrasound instrument on Day 1 and Day 42 after treated with 2 nmol/kg CdSe/ZnS QDs. The detection results and measured parameters were listed in **Figure 6** and **Table 1**, respectively. No significant differences were found in LVAW, LVID, LVPW, and LVV both in end-systolic and end-diastolic of CdSe/ZnS QDs treated mice when compared to the control group. Furthermore, there were also no significant differences in EF, FS, and LV MASS between CdSe/ZnS QDs group and the control group.

**Table 1:** Result of cardiac function measurement (mean ± SD, *n* = 7).

Item	Control	QDs-Day 1	QDs-Day 42
LVAW, d (mm)	1.089 ± 0.126	1.096 ± 0.171	1.122 ± 0.128
LVAW, s (mm)	1.438 ± 0.139	1.419 ± 0.182	1.446 ± 0.147
LVID, d (mm)	3.116 ± 0.286	3.126 ± 0.278	3.142 ± 0.221
LVID, s (mm)	2.218 ± 0.483	2.196 ± 0.414	2.234 ± 0.398
LVPW, d (mm)	1.101 ± 0.207	1.109 ± 0.116	1.069 ± 0.149
LVPW;s (mm)	1.320 ± 0.204	1.307 ± 0.198	1.236 ± 0.167
EF (%)	59.854 ± 12.198	57.149 ± 11.447	56.985 ± 10.709
FS (%)	29.894 ± 10.665	29.801 ± 11.314	28.880 ± 9.695
LV MASS (mg)	95.278 ± 22.945	94.286 ± 25.758	97.840 ± 24.684
LVV; d (µl)	40.383 ± 12.019	41.418 ± 10.698	39.176 ± 11.068
LVV; s (µl)	15.667 ± 3.921	15.881 ± 5.657	16.851 ± 4.881

### Oxidative Stress Markers Detection

**Figure 7:**
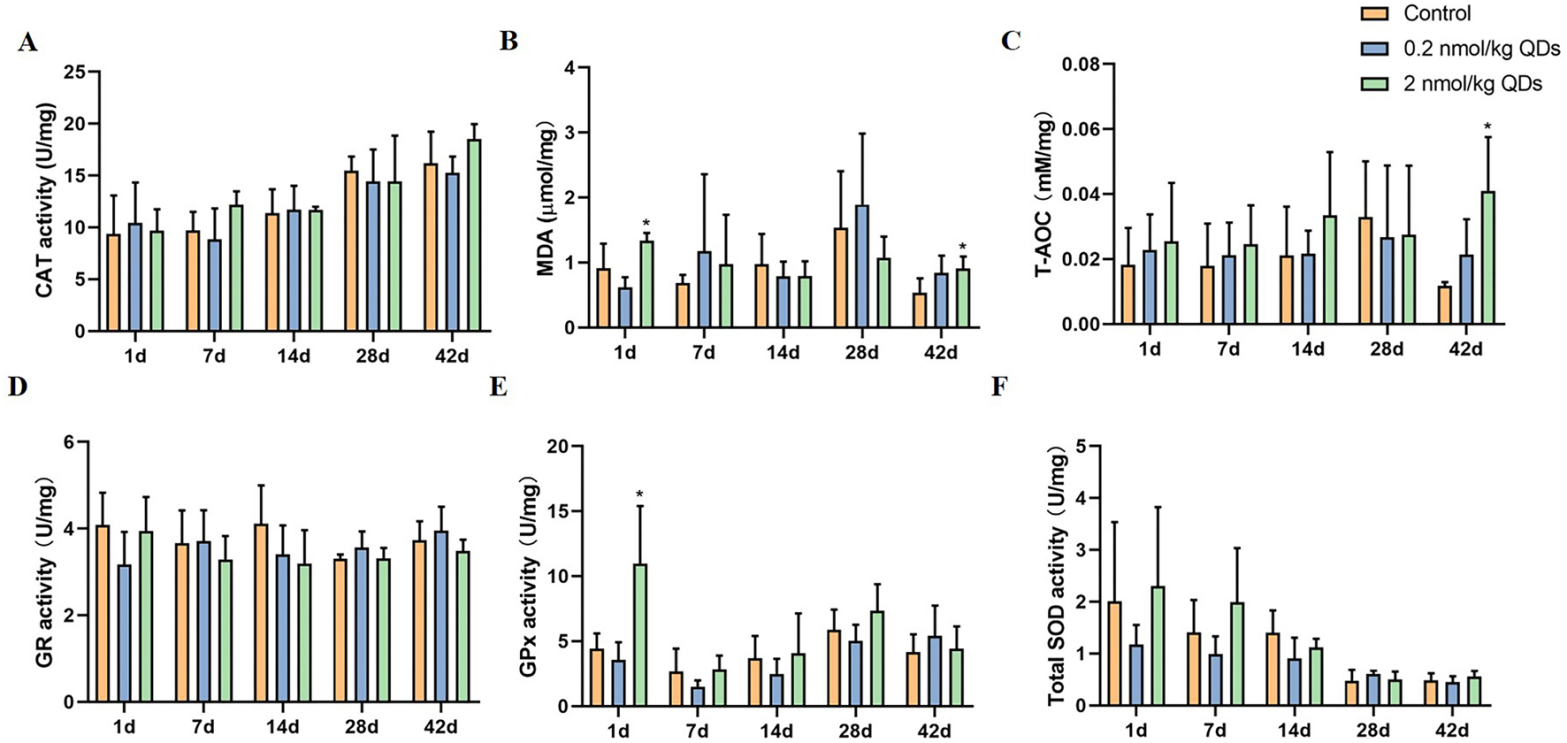
Effects of QDs on oxidative stress in cardiac tissues. **(A)** CAT activity. **(B)** MDA level. **(C)** T-AOC level. **(D)** GR activity. **(E)** GPx activity. **(F)** Total SOD activity. (*Significantly different compared to control *P* < 0.05.).

As CdSe/ZnS QDs could remain in the heart for at least 42 days and caused adverse reactions to the heart, we measured the oxidative stress markers in the heart tissue collected at different sampling times. The activities of CAT, T-AOC, MDA, GPx, GR, and SOD were shown in **Figure 7**. Activities of oxidative stress markers in heart tissue of low dose QDs exposed group showed no significant change at different sampling time when compared with the control group. Activities of MDA and GPx of high dose QDs group were significantly higher than that of the control group on Day 1. Activities of T-AOC and MDA of high dose QDs group were also significantly higher than that of the control group on Day 42. There were no significant changes in other oxidative stress indicators between the high dose group and the control group.

## Discussion

There is no doubt that the use of QDs has provided benefits to light emitting devices, fluorescence imaging, medicine, and medical science, which can be described as revolutionary (Chan et al., 2002). Nevertheless, since these original materials contain heavy metal, such as Cd, As, Pb, and Te, there is an urgent need to understand their potential effects when the QDs were exposed to the environment or accumulated in organisms. The toxicity of QDs has been previously evaluated both *in vitro* and *in vivo*. Studies of cytotoxicity of QDs have shown that QDs could result in reducing cell viability, causing apoptosis, generating ROS, and damaging mitochondria or cell death through a variety of cell experiments (Nagy et al., 2012; Nguyen et al., 2013). However, some studies have revealed that QDs have no obvious effect on cell viability or apoptosis (Peng et al., 2013). This discrepancy may be due to differences in experimental conditions, such as QDs size distribution, element composition, surface modification, exposure dose, interaction time, or cell type (Clift et al., 2008; Brunetti et al., 2013). The *in vivo* toxicity of QDs has also been reported in several systems in some research. QDs can induce persistent inflammation and lung dysfunction in the lung, or damage the liver morphology, and lead to centrilobular fibrosis and oxidative stress in the liver (Haque et al., 2013; Roberts et al., 2013). QDs may also affect embryonic development in zebrafish models (Rotomskis et al., 2018). QDs based on CdSe are one of the most classical, common, and widely used at present. This study was designed to understand the cardiotoxicity of CdSe/ZnS QDs in BALB/c mice from behavioral, body weight, blood biochemistry, histopathology, and echocardiography, and also investigated the oxidative stress mechanism after exposed to QDs.

CdSe QDs have two well-known toxic elements Cd and Se, which can produce harmful effects on cell lines and organisms. Cd is a toxic heavy metal element and potential carcinogen. It can enter the human body by different mechanisms and mainly store in soft tissues with a diversity of toxic effects such as nephrotoxicity, hepatotoxicity, endocrine, and reproductive toxicity. Moreover, Cd can penetrate through the blood-brain barrier and accumulate in the brain. Cd-dependent neurotoxicity is also associated with neurodegenerative diseases such as Alzheimer’s disease and Parkinson’s disease, amyotrophic lateral sclerosis, and multiple sclerosis (Luevano and Damodaran, 2014; Branca et al., 2018). Se is an essential trace element for many species, including humans, and plays a major role in maintaining the normal structure and function of the cardiovascular system (Valdiglesias et al., 2010). However, the demand range of Se in living organism is very narrow, too low of Se can cause selenium deficiency symptoms and too high can also cause toxic effects include arrhythmia, hemolysis, liver necrosis, pulmonary edema, brain edema, and even death (Zwolak and Zaporowska, 2012; Schiavon and Pilon-Smits, 2017). We administered carboxylated CdSe/ZnS QDs in BALB/c mice *via* the tail vein and analyzed the Cd and Se levels in the heart of mice sacrificed by cervical dislocation on Day 42. From the results of ICP-MS, it showed that Cd was detected in the heart of mice exposed CdSe/ZnS QDs, whereas no Cd was detected in the hearts of mice in the control group. The results indicated that the QDs could enter and accumulate in the heart tissue for at least 42 days. However, there was no significant increase of Se level in QDs treated group when compared with that in the control group. Se is an essential trace element in the body and exists in all of the tissues. Although QDs could be accumulated in the heart, Se from CdSe QDs could not significantly increase Se concentration in the heart of mice treated with QDs.

In our previous studies, we found that QDs could be distributed to heart, liver, spleen, lung, and kidney along with the blood transport and can also be distributed to brain or testis through blood-brain barrier and blood-testis barrier (Wang et al., 2016; Zou et al., 2019). The QDs would be metabolized, cleared, or accumulated in tissues finally. Blood routine and blood biochemistry are commonly used indicators of clinical, which can be sensitive to many pathological changes in the body (Yang et al., 2019). When QDs were injected intravenously into mice, they first contacted with the blood. In order to further study the adverse effect of QDs, the hematology parameters including WBC, RBC, and PLT were determined in this study. No significant differences were found in levels of WBC and RBC in CdSe/ZnS QDs treated mice as compared to the control group at different time. Levels of PLT of mice in QDs exposed group increased on Day 1, which indicates that QDs may cause acute infection in mice. LDH, CK, and CK-MB are three commonly used indicators for reflecting the changes of cardiac function. LDH is an important isoenzyme in glycolysis and gluconeogenesis, which widely exists in heart, liver, lung, and many other tissues. When the tissues are damaged, LDH will leak into the blood from organs and then lead to the increase of LDH and its isoenzyme levels in serum (O’Brien, 2008). CK mainly exists in myocardium and skeletal muscle. Increased CK levels may be associated with the damage of heart or brain. It is well known that CK is a valuable characterized marker for cellular damage in a variety of cardiac disease models such as myocardial infarction, viral myocarditis, pericarditis, and cerebrovascular accident (Ibrahim et al., 2019). CK-MB is one of the isoenzymes of CK, which mainly distributes in myocardium. When the myocardium is injured, it will be released into the blood, which leads to the rapid increase of CK-MB level in the blood. In this study, we detected LDH, CK, and CK-MB levels in the serum of mice and found that the levels of LDH, CK, and CK-MB in QDs exposed group were significantly higher than that in the control group on Day 1. CK-MB levels in QDs exposed group were still higher than that in control group on Day 7. It may indicate that QDs caused damage to the heart when exposed through tail vein injection in the early time. After that, myocardial damage recovered as QDs was excreted or degraded over time. The results suggested that CdSe QDs may lead to hematological changes, as they were metabolized and degraded *in vivo* gradually. Prior studies have suggested the stability of the covalently bound CdSe core and the protection of the ZnS shell and surface coating. This Cd complex in the binding state has no valence charge and, therefore, shows no bioreactivity and no obvious toxicity. However, if the CdSe core is degraded, Cd^2+^ will be released, which increases the risk of target organ toxicity (Bertin and Averbeck, 2006; Peng et al., 2013).

Histopathological examination is part of the most commonly used experimental methods in clinical diagnosis, which provides a reliable basis for physiological system diseases. H&E staining was utilized to observe the histopathological changes of the hearts of the mice at different times after injecting QDs. No significant pathological changes were observed. Echocardiography can clearly and noninvasively detect the structural and hemorheological changes of the heart and detect the organic and functional changes of the heart more intuitively. We also measured the echocardiogram of the mice. It was found that there was no statistical difference of the measured parameters between the QDs treated group and the control group. Although CdSe/ZnS QDs can cause some changes in blood biochemical parameters, they do not cause histopathological or echocardiographic changes. These results indicated that QDs have not yet induced pathological and organic changes in the heart of mice.

Oxidative stress is a common mechanism of toxicity induced by many nanomaterials. In order to explore the mechanism of adverse cardiac effects caused by CdSe/ZnS, we analyzed the oxidative stress markers, CAT, T-AOC, MDA, GPx, GR, and SOD, which have often been used to evaluate the oxidative stress status of tissues. In general, SOD is an important peroxidase *in vivo* and can catalyze ·O_2_^-^ into H_2_O_2_. Catalase can catalyze H_2_O_2_ to produce H_2_O and O_2_. T-AOC is the total level of various antioxidants in tissues and reflects the ability of scavenging reactive oxygen species (ROS). GSH scavenges free radicals and some organic peroxides, and GSH redox cycle plays an important role in endogenous antioxidant system *in vivo*. GR can maintain GSH levels in cells and tissues. GSH-Px can specifically catalyze the reaction of reduced GSH with ROS to produce oxidized glutathione GSSG, thus protecting the biofilm from ROS damage and maintaining the normal function of cells (Wang et al., 2017). MDA content is an important parameter reflecting the potential antioxidant capacity of the organism. It can reflect the rate and intensity of lipid peroxidation and indirectly reflect the degree of tissue peroxidation damage. Cardiac GPx and MDA activities of mice in high dose QDs exposed group increased significantly on Day 1. Meanwhile, T-AOC and MDA activities of mice in high dose QDs exposed group also increased significantly on Day 42 when compared with the control group. These results indicated that QDs can cause oxidative damage and produce free radicals when they entered the body. Free radicals react with lipids to produce MDA by peroxidation. Tissues can alleviate peroxide stress by increasing the activity of antioxidant enzymes, so as to quickly restore the balance between oxidation and antioxidation *in vivo* and achieve the purpose of protecting the body.

## Conclusion

In summary, after a single intravenous injection of CdSe/ZnS, animal mortality, hematology, serum biochemistry, histology, heart elements concentration, and echocardiography were investigated at different time after treatment. Cd from QDs could be detected in heart tissues up to 42 days. The PLT count, LDH, CK, and CK-MB of mice exposed to CdSe/ZnS QDs were significantly differences with that of the control group on Day 1, and then returned to normal except CK-MB levels remained high on Day 7. No significant histopathological changes and cardiac function were observed. It revealed that QDs showed cardiotoxicity in mice. To further study the mechanism, we examined some oxidative stress markers in the heart tissues. Activities of GPx and MDA of high dose QDs exposed group were significantly increased on Day 1, and T-AOC and MDA activities of high dose QDs exposed group were significantly increased on Day 42. These results showed that high dose CdSe/ZnS QDs could induce oxidative damage when they entered the body, but tissues can alleviate peroxide stress by increasing the activity of antioxidant enzymes to protect the body from oxidative damage. In summary, we believe that, although this study is relatively simple, it provides important evidence for the biosafety of QDs, especially for the compatibility of the cardiovascular system. In view of the great potential of QDs in biomedical detection, drug delivery, and gene delivery, we hope that more stable and safer QDs can be manufactured and provide great help for future clinical applications of QDs.

## Data Availability Statement

The raw data supporting the conclusions of this manuscript will be made available by the authors, without undue reservation, to any qualified researcher.

## Ethics Statement

The animal study protocols were conducted in accordance with the Guide for the Care and Use of Laboratory Animals Center of Shenzhen University and approved by the Experimental Animal Ethics Committee of Shenzhen University (Permit No.201412012).

## Author Contributions

GL and LL conceptualized and designed the manuscript. XW and GX supported for administrative. LL, JT, DL and WJ experimented and collected the data. ZY analyzed the data. LL drafted and revised the manuscript.

## Conflict of Interest

The authors declare that the research was conducted in the absence of any commercial or financial relationships that could be construed as a potential conflict of interest.
